# Untargeted metabolomic genome-wide association study reveals genetic and biochemical insights into polyphenols of apple fruit

**DOI:** 10.1093/hr/uhaf159

**Published:** 2025-08-12

**Authors:** Jun Song, Beatrice Amyotte, Leslie Campbell Palmer, Melinda Vinqvist-Tymchuk, Kyra Dougherty, Letitia Da Ros

**Affiliations:** Agriculture and Agri-Food Canada, Kentville Research and Development Centre, 32 Main Street, Kentville, Nova Scotia, B4N 1J5, Canada; Agriculture and Agri-Food Canada, Kentville Research and Development Centre, 32 Main Street, Kentville, Nova Scotia, B4N 1J5, Canada; Agriculture and Agri-Food Canada, Kentville Research and Development Centre, 32 Main Street, Kentville, Nova Scotia, B4N 1J5, Canada; Agriculture and Agri-Food Canada, Kentville Research and Development Centre, 32 Main Street, Kentville, Nova Scotia, B4N 1J5, Canada; Agriculture and Agri-Food Canada, Fredericton Research and Development Centre, 95 Innovation Rd, Fredericton, New Brunswick, E3B 4Z7, Canada; Agriculture and Agri-Food Canada, Summerland Research and Development Centre, 4200 Highway #97, South, Summerland, British Columbia, V0H 1Z0, Canada

## Abstract

Apple (*Malus × domestica*) is one of the most popular fruits grown and consumed worldwide, contributing to human health with significant amounts of polyphenols and other bioactive compounds, and providing positive impacts to the economy and society. Understanding the diversity and inheritance of health-active compounds in apple can provide novel selection criteria for future breeding and cultivar development, as consumers increasingly prioritize the health benefits of their food choices. We therefore conducted an untargeted metabolomic analysis using ultra-high-performance liquid chromatography-mass spectrometry (UPLC-MS) to investigate thousands of semipolar chemicals, mainly phenolic compounds, in 439 diverse apple accessions, and quantified 2066 features in positive ion mode. To identify key areas of genetic control for apple metabolite abundance, we performed a metabolomic genome-wide association study (mGWAS) on the quantified mass features using ~280 000 single nucleotide polymorphisms (SNPs). The mGWAS revealed >630 significant loci with hotspots for various groups of known and unknown phenolic compounds including flavonols on Chromosome 1, dihydrochalcones on Chromosome 5, and flavanols on Chromosomes 15 and 16. The most significant hotspot on Chromosome 16 included bHLH and C2H2 transcription factors that may play a role in controlling the abundance and complexity of phenolic compounds through regulation of the flavonoid biosynthesis pathway. Our analysis links the apple metabolome with candidate genes and biosynthetic mechanisms and establishes a foundation for marker-assisted breeding and gene editing to improve and modify phenolic compounds in apple for marketability and the benefit of human health.

## Introduction

Apple is an important fruit crop and has significant impacts on human health and positive socioeconomic benefits. In 2022, the production of apple worldwide reached 96 million tons with a market value of 69 billion dollars [1]. Apple fruit quality attributes are essential drivers of production and consumption, and nutritional content can significantly impact consumers’ perceptions of apple quality [2]. Phenolic compounds are nutritional components found in fruits and vegetables that contribute to human health, nutrition, and medicine due to their antioxidant capacity [3, 4]. In addition to their human health benefits, phenolic compounds play important roles in plant development, defense, and environmental interaction [5]. Phenolic compound abundance in apples is influenced by environmental factors such as climate and ripening stage, as well as by genotype [6]. Wild apple accessions generally have increased levels of phenolic compounds relative to modern cultivars, as improving phenolic content has not historically been a focus of breeding selection [7]. While overall high phenolic content is associated with bitter flavor and astringency, the concentration of select health-active molecules such as certain flavonoids could be increased without detriment to eating quality [8]. Genetic markers associated with specific phenolic compounds could therefore enable breeders to precisely manipulate phenolic content and develop desirable new apple cultivars with improved nutritional value [9].

The genetic architecture of phenolic content in apple has been examined over the last 12 years through both quantitative trait locus (QTL) mapping and genome-wide association studies (GWASs). A major QTL for several phenolic compounds on Chr. 16 was first mapped in biparental dessert apple populations [10, 11]. We previously found the Chr. 16 hotspot to be associated with epicatechin, catechin, and procyanidins B_1_, B_2_, and C_1_ using a high-performance liquid chromatography (HPLC)-GWAS approach [12]. More recently, another GWAS reported significant associations for 30 phenolic compounds, with Chr. 9 being the most important locus [13]. The discrepancies between studies can likely be attributed to population composition. It is therefore crucial that GWAS be conducted in large and diverse populations that are relevant to future breeding objectives. A large GWAS of total phenolic content in cultivars, breeding selections, and diverse accessions recently identified two major loci on Chromosomes 15 and 16 [14].

Mass spectrometry-based metabolomics is a high-throughput method to profile hundreds and thousands of metabolites with quantitation [15]. Metabolomic GWAS (mGWAS) was first applied in Arabidopsis (*Arabidopsis thaliana*) [16], followed by rice (*Oryza sativa*) [17] and maize (*Zea mays*) to detect significant genomic association for dozens to thousands of metabolites [18]. These works demonstrated that combining large-scale metabolomic and genomic data through mGWAS can help to unravel the genetic architecture of metabolic networks [18]. A large-scale apple mGWAS was recently published, examining 2575 metabolites in 270 diverse accessions [19]. Here, a strong peak on Chr. 16 was reported for procyanidins A_2_, C_1_, and B_4_, cinnamtannin D_1_, and arecatannin B_1_; procyanidin A_2_ also had a second strong peak on Chr. 15 [19]. These results concur with the most recent phenolic GWAS in apple [14], but leave open questions related to the roles of other loci such as Chr. 9 [13]. Additional research is therefore required to build a firmer consensus regarding the genetic architecture of the apple phenolic metabolome.

Under the metabolomics framework, untargeted metabolomic analysis has become a method of choice for research requiring comprehensive unbiased ion detection, quantitation, and repeatability [20, 21]. Briefly, untargeted metabolomic analysis provides the opportunity to detect and quantify a nearly comprehensive number of metabolites in a biological sample, while targeted approaches require prior chemical characterization and annotation of the molecules to be detected and quantified [22]. Untargeted metabolomic analysis in mGWAS has been reported only once so far in apple [23]. In this study, the metabolomic phenotypes gathered from ultra-high-performance liquid chromatography–quadrupole time-of-flight mass spectrometry (UHPLC–QTOF-MS) and nuclear magnetic resonance (NMR) spectrometry resulted in 1422 significant marker–trait associations, with Chromosomes 16 and 17 representing the major hotspots [23]. Although this research demonstrated the ability to detect and quantify thousands of metabolomic features simultaneously, no further chemical identification was performed or reported on the detected mass features. Thus, no inferences could be made regarding the role of Chromosome 16 and 17 hotspots in the genetic control of important metabolites such as phenolic compounds.

The objective of this study was to catalogue the chemical diversity and identify putative genetic control of apple polyphenols in a collection of over 400 diverse and breeding-relevant germplasm accessions which had been evaluated for other quality traits by Watts *et al.* (2023) [14]. We applied advanced untargeted metabolomic analysis in combination with mGWAS to further characterize the associations between potentially health-active secondary metabolites and key regions of genetic variation in apple. The results provided novel insight into biosynthesis and regulatory mechanisms, accelerating the discovery of novel candidate genes involved in fruit secondary metabolism. 

## Results

### Characterization and identification of phenolic compounds in apple using an untargeted metabolomics approach

Untargeted metabolomics analysis detected and quantified 2066 and 2500 mass features in positive and negative modes, respectively. This paper examined the features detected in positive mode (Supplementary Tables S1–S3); however, full data for the negative mode can be obtained by contacting the corresponding author. The overall distribution of the quantitative data was normal, and a phenotypic principal component analysis (PCA) using positive-mode data showed that separation of the 439 apple varieties along PC1 (12.3%) and PC2 (7.3%) was likely driven by cumulative metabolite abundance (Supplementary Fig. S1). Cumulative metabolite abundance by apple ranged from 12 600 to 111 600 units of detector response (DRU), with varieties ‘S16-06-72’, a breeding selection from Canada, and ‘Kinsei’, a Japanese cultivar, having the highest and lowest values, respectively (Supplementary Fig. S1). Most of the features had significant differences in abundance due to apple genotype and showed variation across the population. In addition, several features had substantial ranges of abundances. For example, maximum fold-differences of 43x, 91x, and 38x were found for chlorogenic acid (4.82_377.08), epicatechin (5.54_291.09) and phloridzin (8.08_436.14n), respectively (Supplementary Table S2).

Among the analyzed features, 15 were identified with reference standard confirmation based on retention times and masses (Supplementary Table S3). The distributions of reference standard-confirmed features were mostly normal across the population; however, some such as procyanidin C_1_ (5.66_866.2062n), procyanidin B_2_ (5.15_578.1434n), and cyanidin 3-O-beta-galactoside (4.55_449.1086 *m*/*z*) had wide distributions with some skewness (Supplementary Fig. S1). An additional 99 features were putatively annotated based on their mass accuracies within the error of tolerance, their MS fragments, and their isotope similarities, as well as by comparison with prior publications. Information on all feature detections and putative identifications can be found in the supplementary results (Supplementary Table S3). We also conducted additional analysis on ion mobility using the Traveling Wave MS setting to determine the collisional cross section (^TW^CCS_N2_) values and further facilitate the feature classification and identification (Supplementary Method SM1). The ^TW^CCS_N2_ values for reference standards and putatively identified features are available in the supplementary files (Supplementary Table S5).

### Phenolic compounds in apple and their associated genomic hotspots

Metabolomic GWAS performed on the 2066 mass features quantified in positive mode produced a comprehensive view of genomic loci associated with phenolic metabolites in apple. The 439 apples in this study were a representative subset of the larger Apple Biodiversity Collection containing 1119 diverse accessions, as shown by a genomic principle component analysis (Supplementary Fig. S2). Together, these apples represented a diverse population with high levels of phenotypic diversity and a broad range of temporal and geographic origins. The collection included modern cultivars, heritage varieties, numbered accessions from breeding programs, and genebank accessions from wild sources [24].

**Figure 1:**
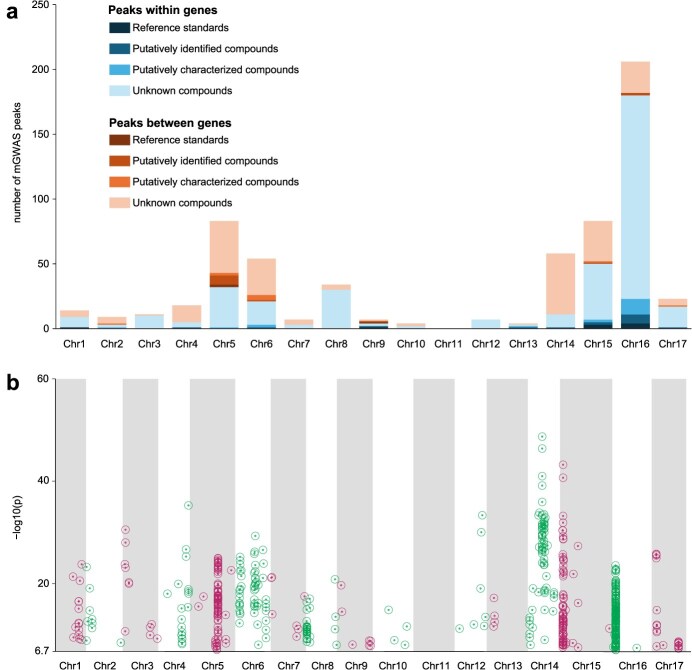
Strong mGWAS peaks for phenolic metabolites in apples: (a) number of peaks by chromosome and (b) *p*-values.

Within this collection, there were a total of 1118 statistically significant associations (*P* < 1.85 × 10^−7^) detected across all 17 chromosomes. Many were observed to have scattered single nucleotide polymorphisms (SNPs) without cohesive structure on the Manhattan plots and quantile–quantile plots indicating weak model fits, and were therefore excluded from further analyses (data not shown). In contrast, 632 strong peaks were identified in which markers converged into a narrow column at a specific genomic position on the Manhattan plots (Fig. 1a, Supplementary Table S4). Among these strong peaks, 75% (473) were found to lie within genomic hotspots: loci for which the most significant SNP was conserved across at least three metabolomic features (Fig. 1b). Overall, the most intensive hotspots were found on Chromosomes 5, 14, 15, and 16; together the 33 hotspots across these chromosomes represented the most significant associations for 59% (374) of the mass features. The largest hotspot was Chr. 16 position 3,421,803 (*16_3,421,803*), which was the most significant SNP within a strong peak for 18% (115) of the metabolites. Other hotspots of note included *16_3,178,627* (34 metabolites); *15_3,168,243* (30 metabolites); *5_29,703,521* (24 metabolites); and *14_14,202,308* (15 metabolites) (Fig. 1a, Supplementary Table S4). For 23 of the hotspots, including *16_3,421,803*, *16_3,178,627*, and *15_3,168,243*, the top SNP was found to be located within a gene (Table 1).

**Table 1:** Summary of mGWAS hotspots for which the top SNP is located in, or near, a gene. Major candidate genes of interest are in bold and nonsynonymous SNPs are underlined

	SNP				Major		Minor		Associated compounds	
Chr.	Position	Gene^a^	Location in gene	Annotation	Allele	AA^b^	Allele	AA	No.	Examples^c^
1	24,595,544	MD01G1136700 (−2)	Exon	RING-H2 finger C2A	T	R	C	R	4	Quercitrin (7.38_448.1016n)^1^
3	3,153 ,356	MD03G1039800 (+1)	Exon	Transducin/WD40 repeat-like superfamily protein	G	T	C	T	3	All unknown
5	27,046,840	MD05G1139700 (+)	Exon	Glutamate receptor 2	G	R	C	P	3	All unknown
5	29,523,731	MD05G1166000 (+)	Downstream of gene	Cellulose-synthase-like C6	T		G		7	All unknown
5	29,553,178	MD05G1166100 (+)	Exon	Ankyrin repeat family protein	C	L	T	F	10	2-Furylmethyl (2E)-2-butenoate (8.10_149.0599 m/z)^3^
5	29,700,524	MD05G1166800 (+1)	Exon	Oxidoreductase	A	S	G	S	3	All unknown
6	4,710 ,729	MD06G1037100 (−)	3’ Untranslated region (UTR)	SNARE associated Golgi protein family	C		T		7	All unknown
6	20,565,071	MD06G1084100 (+)	Intron	Tyrosyl-tRNA synthetase	A		G		8	Rehmaionoside C (7.13_371.2069 m/z)^3^ Eriojaposide A (7.13_502.2420n)^3^
8	470,193	MD08G1005200 (+)	Intron boundary	Enoyl-CoA hydratase 2	C		G		22	All unknown
14	1,148 ,583	MD14G1012400 (−)	Intron	ELMO/CED-12 family protein	A		G		5	Trans-5-O-(4-coumaroyl)-D-quinic acid (5.71_338.1006n)^2^
14	15,995,981	MD14G1103600 (−)	Intron	Polynucleotidyl transferase	A		T		8	All unknown
**15**	**3,168 ,243**	**MD15G1046100 (−)**	**Intron**	**Non-specific serine/threonine protein kinase**	**T**		**C**		**30**	**Epicatechin (5.54_290.0795n)** ^**1**^ **Procyanidin C1 (5.66_866.2062n)** ^**1**^
15	3,360 ,112	MD15G1049300 (−)	Intron	Leucine-rich receptor-like protein kinase family protein	C		A		4	All unknown
15	3,918 ,549	MD15G1058500 (+)	Intron	FTSH protease 4	G		A		7	Kaempferol 3-(3″-p-coumarylglucoside) (6.65_577.1346 m/z)^2^
16	3,161 ,430	MD16G1044700 (+3)	Exon	Regulator of Vps4 activity in the MVB pathway protein	T	T	C	T	4	All unknown
**16**	**3,178 ,627**	**MD16G1045300 (+)**	**5’ UTR**	**Aluminum activated malate transporter family protein**	**A**		**G**		**34**	**Procyanidin A2 (4.40_576.1270n)** ^**1**^
16	3,248 ,848	MD16G1046800 (−)	5’ UTR	Phytochrome and flowering time regulatory protein (PFT1)	G		T		5	All unknown
16	3,304 ,530	MD16G1047400 (+1)	Exon	C2H2-like zinc finger protein	C	R	A	R	4	Procyanidin B1 (4.36_578.1430n)^1^
16	3,319 ,009	MD16G1047600 (−1)	Exon	Methionine aminopeptidase 1B	C	S	A	I	4	All unknown
**16**	**3,421 ,803**	**MD16G1048700 (−1)**	**Exon**	**Basic helix–loop–helix (bHLH) DNA-binding superfamily protein**	**T**	**K**	**C**	**K**	**115**	**Procyanidin B2 (5.15_578.1434n)** ^**1**^ **Epicatechin (5.54_290.0795n)** ^**1**^ **Procyanidin C1 (5.66_866.2062n)** ^**1**^
17	4,222 ,397	MD17G1054600 (−)	Intron	Chorismate mutase 1	A		T		4	Quercetin 3-O-malonylglucoside (7.08_550.0967n)^2^
17	4,752 ,203	MD17G1058200 (−)	Exon	UDP-Glycosyltransferase superfamily protein	T	Q	C	R	3	All unknown
17	27,320,400	MD17G1224700 (−)	Intron	Protein phosphatase 2C family protein	A		T		11	All unknown

a Gene names, annotations, and coding sequences obtained from the GDDH13 v1.1 reference genome^46^ on GDR [25]; symbols indicate forward (+) or reverse (−) coding strand. For SNPs located in exons, reading frame is given numerically (e.g. +1 = 1^st^ forward reading frame).

b AA = putative amino acid encoded by the codon including the major or minor SNP allele.

c Numerical annotations indicate level of compound identification as (1) identified, (2) putatively identified, and (3) putatively characterized.

Due to the complex nature of the polyphenols in apple, our results primarily focused on the compounds confirmed with reference standards (Supplementary Table S3). A total of 12 strong peaks were identified for reference standard-confirmed compounds, nine of which had their most significant SNPs located within genes (Fig. 1a). The peaks within genes included the hotspot at *16_3,421,803*, which was associated with procyanidin B_2_, procyanidin C_1_, and epicatechin (Table 1, Supplementary Table S4). Overall, the majority of peak SNPs (63%, 395 peaks) were found to lie within gene coding sequences (Fig. 1a). For putatively identified and characterized compounds, a total of 33 peaks were found within genes and 23 between genes, i.e. in noncoding regions. Chromosomes 5, 6, 9, 15, and 16 were the most important genomic regions for reference standard-confirmed, putatively identified, and characterized compounds. In contrast, several unknown compounds were associated with Chr. 14 and very few were associated with Chr. 9. It was relatively rare for metabolites to be associated with more than one genomic locus; only 11% (61) of metabolites had two strong peaks in the mGWAS while the rest (89%, 510) had single peaks. The overall largest category of strong associations (28%, 179 peaks) were those located within genes on Chr. 16 (Fig. 1, Supplementary Table S4).

### Phenotypic relationships among compounds associated with major genomic hotspots

**Figure 2:**
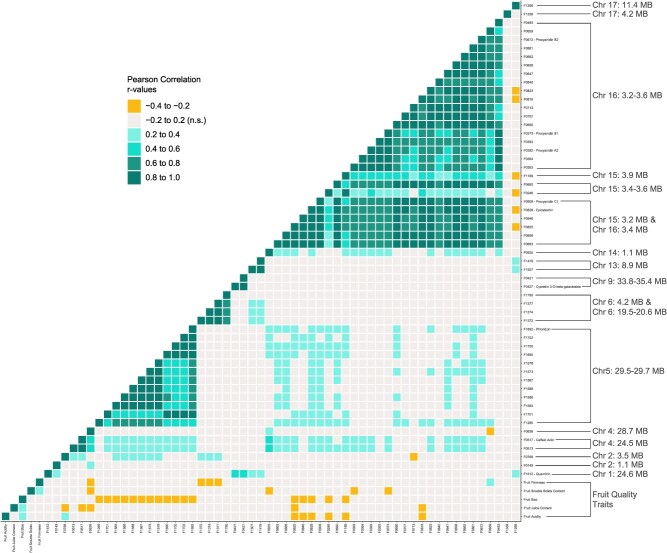
Pearson correlations for fruit traits and metabolites with the most significant mGWAS regions annotated. Feature abbreviations defined in Supplementary Table S6.

A correlation analysis was performed using compound abundance data for the features with putative identifications (Supplementary Table S3: levels 1 and 2) and strong mGWAS peaks. Basic fruit quality traits from a previous study of the same apples [24] were included to evaluate their relationships with phenolic compound abundance. Overall, positive correlations were found among features with common chemical structures (Fig. 2, Supplementary Tables S3, S6). In addition, the chemically related compounds tended to be associated with the same genomic regions (Fig. 2, Supplementary Tables S4, S6). The largest block of positive correlations was observed for the 27 features associated with Chr. 15 and Chr. 16. These included epicatechin (F0828) and procyanidins A_2_ (F0392), B_1_ (F0373), B_2_ (F0672), and C_1_ (F0909). The next largest block was for 12 features associated with Chr. 5 such as phloridzin (F1692) and a phloretin glucoside (F1578); several of these were also positively correlated with epicatechin and other metabolites from the Chr. 15–16 group. A cluster analysis of compound abundances similarly showed that groups of chemically related compounds tended to share genomic hotspots (Supplementary Fig. S3). There were relatively few negative correlations between compounds; however, weak negative correlations were observed between the phenolic metabolites and fruit quality traits. In general, fruit size was negatively correlated with the Chr. 5 and Chr. 15 groups of metabolites, while acidity was negatively correlated with the Chr. 15 compounds. Fruit firmness was negatively correlated with three compounds from the Chr. 6 group. The only positive correlations for fruit quality traits were observed between 1-O-feruloyl-beta-D-glucose (F0768) and acidity, and between 3-methylene-3H-indole (F0659) and fruit firmness (Fig. 2, Supplementary Table S6).

### Chromosome 16 is a major hotspot for phenolic metabolites

**Figure 3:**
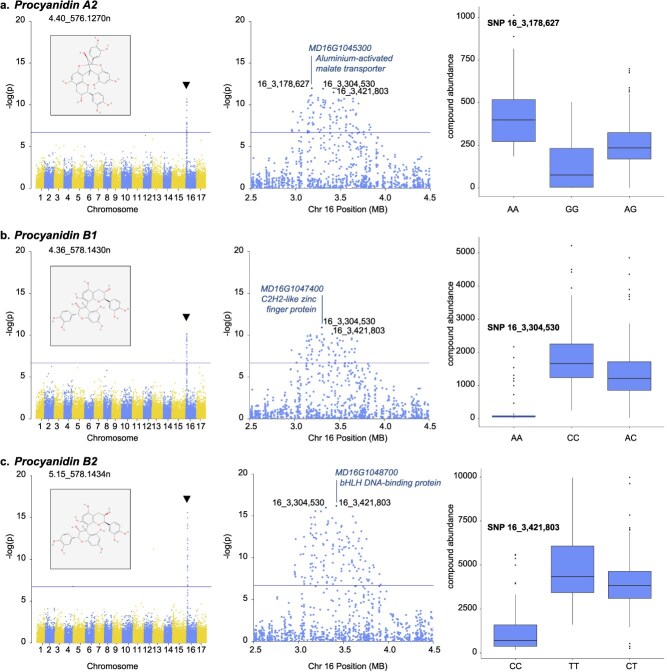
mGWAS results of select phenolic compounds on Chromosome 16, including genome-wide and zoomed-in Manhattan plots, as well as boxplots for the most significant SNPs in association with compounds (a) Procyanidin A_2_, (b) Procyanidin B_1,_ and (c) Procyanidin B_2._ Solid lines on Manhattan plots indicate the significant threshold at *P =* 1.85 × 10^−7^. Chemical structures obtained from PubChem [26]. Gene positions and annotations obtained from the GDDH13.1 reference genome [27] on the GDR [25].

Untargeted metabolomic analysis paired with feature identification provided a detailed view of the chemical composition and diversity of phenolic compounds in apple. Applying these phenomic data to mGWAS revealed a major candidate regulatory region on Chr. 16. In this study, a total of 205 metabolomic features were associated with loci on Chr. 16. Among them, 87% (179) had their most significant SNPs hit directly inside genes (Fig. 1a, Supplementary Table S4). Some genic regions represented more features than others. The largest hotspot was found at position *16_3,421,803* (115 peaks), within the coding sequence for *MD16G1048700*, a basic helix–loop–helix (bHLH) DNA-binding superfamily protein (Table 1). The second largest hotspot was *16_3,178,627* (34 peaks), located within gene *MD16G1045300*, an aluminum-activated malate transporter (ALMT) family protein. These loci were both positioned within a 1-Mb region on Chr. 16 (3.0–4.0 Mb) that was associated with 35% (201) of the mapped metabolites in this study (Table 1, Fig. 3, Supplementary Table S4).

The Chr. 16 hotspots were associated with distinct metabolites. The *16_3,421,803* SNP within *MD16G1048700* (*bHLH*) was the most significant marker for procyanidin B_2_ (5.15_578.1434n), epicatechin (5.54_290.0795n), and procyanidin C_1_ (5.66_866.2062n), together with 112 other metabolites (Fig. 3c, Supplementary Table S4). The putatively identified and characterized compounds associated with this locus included shikimic acid (5.15_139.0395 m/z), eriodictyol (5.61_289.0713 m/z), and 2-hydroxynaringenenin (5.56_311.0531 m/z), among others. The T/C SNP at this locus represented a codon change from AAA to AAG on the reverse coding strand of *MD16G1048700*, causing no change in amino acid as both alleles coded for lysine (Table 1**,**  Supplementary Table S4). Nevertheless, we observed that an allele difference of CC versus TT at *16_3,421,803* was associated with a 3.5-fold increase in abundance (1391 vs 4938 DRU) of procyanidin B_2_ (Fig. 3c). The *16_3,178,627* SNP within *MD16G1045300* (*ALMT*) was the most significant marker for procyanidin A_2_ (4.40_576.1270n) and 33 other metabolites (Fig. 3a, Supplementary Table S4). The A/G SNP at this locus was positioned in an intron and did not cause a putative change in amino acid (Table 1, Supplementary Table S4). However, an allele difference of AA versus GG at *16_3,178,627* was associated with a 30% decrease in abundance (420 vs 128 DRU) of procyanidin A_2_ (Fig. 3a). A third locus of note on Chr. 16 was *16_3,304,530*, which was positioned within *MD16G1047400*, a C2H2-like zinc finger protein. This was the most significant SNP for procyanidin B_1_ (4.36_578.1430n) and three other metabolites (Fig. 3b, Supplementary Table S4). The C/A SNP at this locus represented a codon change from CGG to AGG on the forward coding strand of *MD16G1047400*, a synonymous substitution in which both variants coded for arginine. An allele difference of CC versus AA at *16_3,304,530* was associated with a 5.7-fold increase in abundance (316 vs 1802 DRU) of procyanidin B_1_ (Fig. 3b).

### Chromosome 5 is important for dihydrochalcones

**Figure 4:**
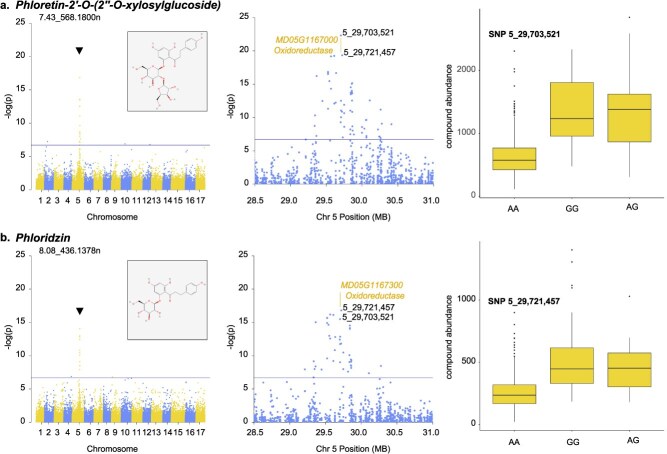
mGWAS results of select phenolic compounds on Chromosome 5, including genome-wide and zoomed-in Manhattan plots, as well as boxplots for the most significant SNPs in association with compounds, (a) phloretin-2'-O-(2"-O-xylosylglucoside) and (b) phloridzin. Solid lines on Manhattan plots indicate the significant threshold at *P =* 1.85 × 10^−7^. Chemical structures obtained from PubChem [26]. Gene positions and annotations obtained from the GDDH13 v1.1 reference genome [27] on the GDR [25].

Significant hotspots of genetic control were also found on Chr. 5 with a total of 70 strong peaks across nine hotspots. Similar to Chr. 16, the majority of the associations on Chr. 5 (87%, 61 peaks) were observed within a single window of 1.5 Mb (29–30.5 Mb) (Fig. 1b, Supplementary Table S4). The largest hotspots were at 5_29,703,521 (24 peaks) and 5_29,721,457 (11 peaks). These were the most significant SNPs for phloretin-2'-O-(2‘-O-xylosylglucoside) (7.43_568.1800n) and phloridzin (8.08_436.1378n), respectively, among other putatively identified dihydrochalcone-type compounds. An allele difference of AA versus GG at 5_29,703,521 was associated with a 2.1-fold increase in abundance (642 vs 1355 DRU) of phloretin-2'-O-(2’-O-xylosylglucoside) (Fig. 4a). Similarly, an allele difference of AA versus GG at 5_29,721,457 was associated with a 2.1-fold increase in abundance (261 vs 546 DRU) of phloridzin (Fig. 4b). Although neither marker was positioned within a gene, both were proximal to a cluster of five oxidoreductase genes including *MD05G1167000* (5_29,703,900…29,704,886) and *MD05G1167300* (5_29,717,650…29,718,779).

### Additional loci of interest for phenolic compound content in apple

**Figure 5:**
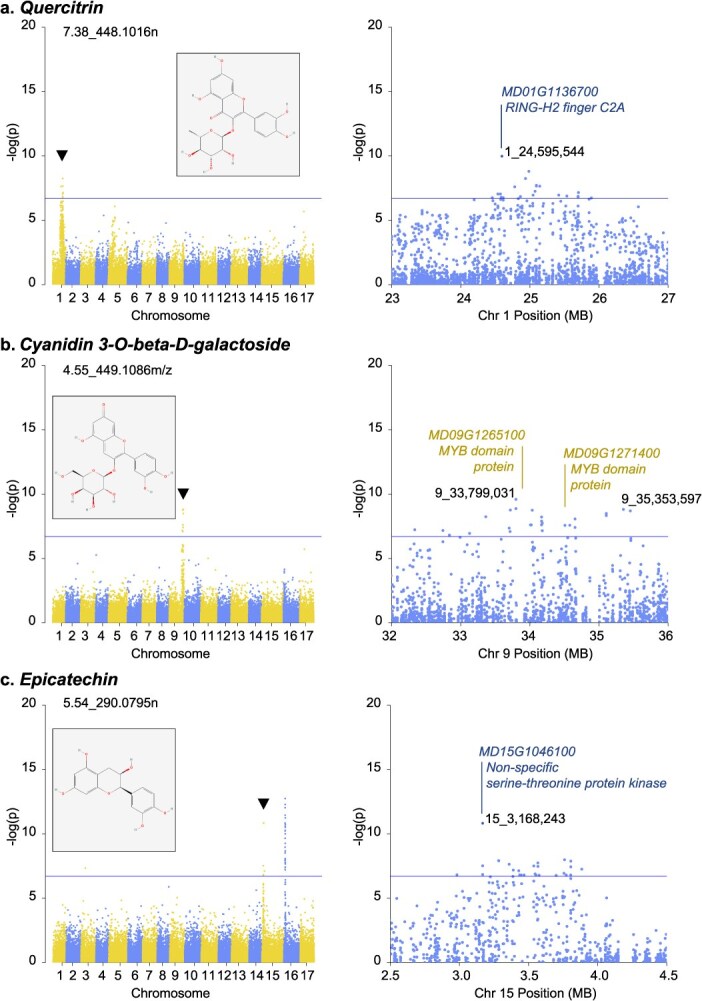
mGWAS results of select phenolic compounds on other chromosomes, including genome-wide and zoomed-in Manhattan plots for compounds (a) quercitrin, (b) cyanidin 3-O-beta-D-galactoside, and (c) epicatechin. Solid lines on Manhattan plots indicate the significant threshold at *P =* 1.85 × 10^−7^. Chemical structures obtained from PubChem [26]. Gene positions and annotations obtained from the GDDH13 v1.1 reference genome [27] on the GDR [25].

Other loci of note varied across compound types. A group of flavonol-related compounds was detected and identified with reference standards, including quercitrin (quercetin-3-rhamnoside; 7.38_448.1016n), isoquercitrin (quercetin 3-O-glucoside; 6.68_464.0964n), avicularin (quercetin-3-O-arabinoside; 7.25_434.0857n), and rutin (quercetin 3-O-rutinoside; 6.46_610.1542n) (Supplementary Tables S2a, S3). These compounds contain 3-hydroxyflavone backbones with flavonol glycosides. Among these, only quercitrin (7.38_448.1016n) had a significant peak in the mGWAS and was associated with *1_24,595,544* (Fig. 5a, Supplementary Table S4). This Chr. 1 SNP was located within gene *MD01G1136700*, which encodes a RING-H2 finger C2A protein (Fig. 5a). There were no strong associations detected for isoquercitrin, avicularin, or rutin, despite these compounds having similar abundances and phenotypic distributions as other mapped compounds across the apple population (Supplementary Fig. S1). An anthocyanin, cyanidin 3-O beta-D-galactoside (4.55_449.1086 m/z), was found to be associated with two peaks on Chr. 9, one at *9_33,799,031* and the other at *9_35,353,597* (Fig. 5b, Supplementary Table S4). The latter of these was located within *MD09G1276200*, an adenine nucleotide alpha hydrolase. In addition, the genomic region between the top markers contained two *MYB*-domain genes, *MD09G1265100* (*9_33,927,995...33,930,960*) and *MD09G1271400* (*9_34,570,103...34,572,449*) (Fig. 5b). While only 11% (61) of the metabolites with significant mGWAS results in this study had two strong peaks, nearly half of these (44%, 27 metabolites) were associated with hotspot loci on Chr. 15 + Chr. 16 (Supplementary Table S4). In total, there were 83 strong peaks detected on Chr. 15, including six hotspots. The largest hotspot was at position *15_3,168,243* (30 peaks) (Table 1). This SNP was located within *MD15G1046100*, a nonspecific serine/threonine protein kinase (STK). Both epicatechin and procyanidin C_1_ were associated with this marker as well as with *16_3,421,803*, the largest hotspot on Chr. 16 (Table 1, Fig. 5c, Supplementary Table S4).

## Discussion

Polyphenols or phenolic compounds are major secondary metabolites that play a significant role in plant development [5] and are beneficial to human health [3]. Untargeted metabolomics analysis enables hundreds to thousands of phenolic compounds in plant products to be simultaneously detected and quantified [20]. Our untargeted metabolomics study quantified >2066 and tentatively identified >100 mass features in ~439 diverse apples, using accepted internal standards and quality control procedures for LC–MS analysis. This quantitative procedure proved to be effective with good repeatability of the method showing a replicate standard deviation of <5% (Supplementary Table S1, Supplementary Fig. S4). We applied these phenomic data in a large-scale mGWAS and detected >630 strong genomic associations for unknown, putatively identified, and reference standard-confirmed phenolic compounds.

The current study improves our understanding of the genotype–phenotype relationships underlying phenolic content in diverse apples and reveals new insights into the major genetic loci controlling synthesis and accumulation of these important health-active compounds. Previous studies investigating the genetic architecture of phenolic content in apple have largely confirmed Chr. 16 as a hotspot for genetic regulation of phenolic compounds. The major publications either paired HPLC with QTL analysis or GWAS [10, 12, 13, 28], or used LC–MS to conduct similar analyses [19, 23, 29]. However, their results revealed a gap in consensus regarding the specific Chr. 16 loci involved, information that is required to elucidate the causal genetic control mechanisms. Our research increases both the scale and precision of metabolomic QTL detection over what was previously reported.

As with all GWAS results, the significant associations reported in this study may not be the polymorphisms directly influencing the phenotypes. Instead, they may simply be in linkage disequilibrium with the true functional genes or regulatory elements. However, this study involved a high density of marker coverage and consistently detected several major hotspot loci using stringent thresholds for marker significance. We therefore propose consideration for the select candidate genes and putative causal SNPs discussed below.

### Flavanols

Flavanols and their polymerized forms including proanthocyanidins and anthocyanidins are the dominant phenolic compounds in apple [30]. These compounds are associated with human health benefits such as antioxidant, antidiabetic, and cardiovascular protective effects [31], and also carry complex bitter flavors [32]. QTLs for flavanols and proanthocyanidins have previously been reported on Chr. 16 [28, 33]. Our current study provides new evidence that flavanol-related compound abundance may be influenced by multiple regions of genetic control on Chr. 16.

We identified a major candidate regulatory region from 3.0 to 4.0 Mb on Chr. 16 that encompassed more than one-third of the marker–trait associations reported in this mGWAS study. This 1-Mb region has fairly conserved linkage disequilibrium (Supplementary Fig. S5), suggesting that one or more gene therein is involved in flavanol regulation. We refer to this region as a megahotspot for phenolic compound content in apple. Looking within the megahotspot, we found peak SNPs for flavanol-related compounds within genes *MD16G1045300*, *MD16G1047400*, and *MD16G1048700*, encoding an *ALMT*, a C2H2-like zinc finger protein (C2H2), and a basic helix–loop–helix DNA-binding superfamily protein (bHLH), respectively (Fig. 2).

The three Chr. 16 candidate genes identified in this study may play functional roles in the regulation of flavanol and proanthocyanidin abundance. *ALMTs* are a family of membrane protein functioning as vacuolar malate transporters and serving to maintain cell malate homeostasis [34]. The *ALMT* (*MD16G1045300*) identified in this study was *Ma1*, a gene previously reported to control acidity in apple fruits [35] (gene formerly listed as *MDP0000252114*). *Ma1* has been shown to regulate the malate content in apple: a premature stop mutation at 1455 bp leads to a truncated protein with reduced malate transport activity, causing low fruit acidity [35, 36]. In our study, the SNP at position *16_3,178,627* was positioned in an intron, and was associated with a 30% difference in procyanidin A_2_ abundance between the homozygous allele classes (AA vs GG) at this SNP, suggesting it may be a causal mutation.

The functional relationship between malic acid membrane transport and flavanol-related compound accumulation remains to be examined. We did not detect a significant correlation between fruit acidity and procyanidin A_2_, suggesting some complexity in the regulatory relationship among these metabolites. SNP markers in *Ma1* were previously found to be associated with fruit acidity in the same apple collection that was used for the present study. However, that study did not find that *Ma1* was associated with total phenolic content [14]. An earlier biparental mapping study did report a shared QTL on Chr. 16 for acidity and phenolic compounds but demonstrated that this overlap did not result from correlation between the traits [29]. Another study identified a gene network of receptor kinases, protein kinases, and *C2H2* transcription factors involved in the regulation of malate content in apples [37]; these genes could perhaps play a larger role in the regulation of acids and phenolic compounds.

C2H2 transcription factors are master regulators of plant development and environmental response [38]. The *C2H2* (*MD16G1047400*) identified in this study has not previously been described in the context of apple transcriptional regulation, although other apple C2H2s have been reported to serve in abiotic stress response [39]. The SNP at position *16_3,304,530* within *MD16G1047400* represented a putative synonymous substitution but was associated with a 5.7-fold difference in abundance of procyanidin B_1_ between homozygous allele classes (CC vs AA). Although not causative, this mutation may be tightly linked to proximal mutations affecting the C2H2 protein expression or function. Comparative sequencing of the *MD16G1047400* gene region could reveal candidate functional mutations linking this specific *C2H2* more directly to flavanol-related compound abundance. C2H2-domain containing genes have been reported to increase in expression during apple fruit ripening [40], although they are not commonly considered to be major factors driving the ripening process [41].

The bHLH DNA-binding superfamily proteins are transcription factors known to regulate flavonoid biosynthesis in plants [42]. In apple, bHLH transcription factors are differentially expressed across organs [43], and have been shown to control leaf shape through auxin signaling as well as fruit ripening through various signaling pathways [44]. One of the earliest QTL studies of phenolic content in apple suggested bHLH genes on Chr. 16 as candidate regulators of phenolic compound abundance [29]. The *bHLH* (*MD16G1048700*) identified in this study represented the single largest mGWAS hotspot with 115 strong associations. The SNP at position *16_3,421,803* within this gene was synonymous and therefore not causal, but we observed a 3.5-fold difference in procyanidin B_2_ abundance between the homozygous allele classes (TT vs CC) at this SNP. Given the substantial number of phenolic metabolites associated with this specific genomic locus, the role of *MD16G1048700* merits further investigation.

The Chr. 16 megahotspot also contained gene *LAR1* (Leucoanthocyanidin reductase 1), a previously reported candidate gene for phenolic content in apple [33, 45]. *LAR1* is proposed to catalyze the synthesis of catechin and epicatechin [45]. The original reported *LAR1* gene *MDP0000171928* aligns with *MD16G1048500* in the GDDH13 v1.1 reference genome [27]. *MD16G1048500* is a NAD(P)-binding Rossmann-fold superfamily protein encoded at Chr. 16 position 3,404,867–3,409,858 [25]. There was no marker within *MD16G1048500*; however, it was positioned within the linkage disequilibrium window of *16_3,421,803* (Supplementary Fig. S5) which was the most significant SNP in this study. Further investigation is required to determine whether expression of *LAR1* is regulated by the transcription factors in this region.

The flavanol-related compounds epicatechin and procyanidin C_1_ were associated with the *bHLH* locus on Chr. 16 as well as with a second locus at 3.17 Mb on Chr. 15 (Fig. 3c). Previous studies have reported QTLs on Chr. 15 for procyanidins and flavanols [28] as well as for total phenolic content [14]. The peak SNP identified in this study was positioned within a nonspecific *STK*. STKs have wide-ranging roles in plant cell immunity and have been proposed as candidate genes for resistance to apple scab [46] and fireblight [47]. The potential for co-regulation of phenolic metabolism between transcription factors on Chr. 16 and signaling genes on Chr. 15 merits further exploration.

### Dihydrochalcones

Dihydrochalcones are an important class of phenolic compounds that are uniquely abundant in apple [48]. They have been reported to function in abiotic and biotic stress responses across a range of apple tissues and to mediate hormone signals driving plant growth and development [49]. For human consumption, these compounds represent a significant source of dietary antioxidants [48]. In this study, we detected and identified or putatively identified the dihydrochalcone compounds phloretin, phloridzin, and phloretin-2'-O-(2"-O-xylosylglucoside) within the diverse apple population (Supplementary Table S3). Significant associations for these compounds were detected on Chr. 5 at 27.7 Mb (Fig. 3). Previous genetic mapping papers have reported variable results for dihydrochalcones in apple fruits. Either there were no QTLs detected [10], QTLs on Chr. 16 [33], QTLs on multiple chromosomes [28], or scattered GWAS hits across the genome [13]. Only one study reported QTLs on Chr. 5 for phloridzin and phloretin xyloglucoside [28].

Although there was no consensus QTL for dihydrochalcones detected across previous publications, Chr. 5 was found to represent an important mGWAS hotspot in the present study. A total of 67 compounds including phloretin, phloridzin, and phloretin-2'-O-(2"-O-xylosylglucoside) had peak SNPs between 29.0 and 30.5 Mb on Chr. 5. The hotspot locus at 27.7 Mb on Chr. 5 was positioned within a region encoding five clustered oxidoreductase genes that may play a functional role in dihydrochalcone accumulation. Phloretin and phloridzin are reportedly synthesized in three steps: (1) reduction of 4-coumaroyl-CoA to 4-dihydrocoumaroyl-CoA by a double-bond reductase, (2) condensation of 4-dihydrocoumaroyl-CoA to phloretin using three molecules of malonyl-CoA by chalcone synthase, and (3) glycosylation of phloretin at the 2′ or 4′ position of ring A to form phloridzin or trilobatin by a UDP-glycosyltransferase [50]. The Chr. 5 oxidoreductases could potentially catalyze the first step of this biosynthetic pathway by functioning as double-bond reductases.

While the Chr. 5 mGWAS peaks do not fall within gene coding sequences, they may be associated with regulatory elements controlling overall expression of the oxidoreductase cluster. Other enzymes in the biosynthetic pathway including a UDP-glycosyltransferase on Chr. 5 and a chalcone synthase on Chr. 15 were previously proposed as candidate genes for dihydrochalcone abundance in apple [28]. Genes encoding these enzyme classes are found throughout the apple genome [27], which may be part of the reason for which the QTLs detected for dihydrochalcones have varied considerably across mapping studies.

### Flavonols

Flavonols and flavonol glycosides are another important group of phenolic compounds in apple with benefits to plant and human health [31], and strongly bitter flavors [32]. QTLs for flavonol glycosides have previously been reported on Chr. 1 [12, 13] and our results identify a new potential gene of interest for future study. The peak SNP for quercitrin and three unknown compounds was located within gene *MD01G1136700*, encoding a RING-H2 finger C2A (RING-H2) protein (Fig. 4a). RING-H2 finger proteins are E3-ubiquitin ligases that play significant roles in plant growth, stress response, and signal transduction [51]; they are also involved in anthocyanin regulation in apple [52]. The SNP at position *1_24,595,544* was located within the *RING-H2* gene *MD01G1136700*. The locus previously reported for quercitrin was on Chr. 1 at 25.7 Mb (McClure *et al*., 2019), which was within the window of significance for the mGWAS peak in this study (Fig. 4). In comparison, the associations previously reported for quercetin-3-O-rhamnoside were further down the chromosome at 26.5 and 27.5 Mb [13]. Flavonoid biosynthesis genes including a UDP-glycosyltransferase and two chalcone-flavone isomerases were proposed as candidate genes by McClure *et al*., (2019) and Kumar *et al*., (2022) [13], respectively. The considerable distances between Chr. 1 loci detected for flavonol glycosides across separate studies suggests that additional research into this genomic region is required. In our study, there were 10 unknown compounds with peak SNPs located between 24.5 and 27.7 Mb; these may also be flavonol glycosides controlled through related mechanisms (Supplementary Table S4).

### The roles of transcription factors

The results of our study support the hypothesis that genetic variation in transcription factors contributes substantially to phenolic diversity in apple. The major finding of this research was the megahotspot on Chr. 16 that contained C2H2 and bHLH transcription factors and was associated with 200 metabolites including flavanols, proanthocyanidins, and many unknown phenolic compounds (Figs 1 and 2).

In connection with recent literature, our results provide strong evidence for the central role of transcription factors in regulating secondary biosynthetic pathways in apples. MYB and NAC family transcription factors on chromosomes 9 and 3, respectively, have been shown to underlie major QTLs for apple color and ripening [14, 53]. In our study, the mGWAS peak for the anthocyanin cyanidin-3-O-beta-D-galactoside was also found on Chr. 9 in proximity to two *MYB*-domain genes (Fig. 4). MYB and NAC transcription factors are involved in upregulating flavonoid pathway genes including chalcone synthases and isomerases, flavonoid hydroxylases and reductases, anthocyanin synthases and UDP-glycosyltransferases [54]. MYB and bHLH transcription factors have specifically been shown to form complexes with a third transcription factor, WD40, to regulate anthocyanin biosynthesis in strawberry and the same mechanism has been proposed in apple [55]. Detailed studies of apple acidity have suggested C2H2 transcription factors may be part of the network regulating malate accumulation via the ALMT malate transporter [37]. The largest mGWAS hotspot in this study contained *bHLH*, *C2H2*, and *ALMT* genes (Fig. 2). While we did detect some candidate genes putatively involved in biosynthesis (Fig. 3), our results suggest that the genetic control of apple phenolics lies primarily at the network level. The specific *C2H2* and *bHLH* genes identified in this study merit further investigation so that their precise roles in the apple phenolic regulatory network can be verified and characterized.

### Marker-assisted selection

Despite the need for functional genetics studies to validate the candidate genes detected in this study, there is an immediate opportunity to apply the peak SNPs in marker-assisted selection due to their significant statistical associations with phenolic compound abundance in apple. The 5.7-fold difference in quantitative phenotypes between allele classes supports the use of the *16_3,304,530* SNP marker for proanthocyanidins and other flavanols. Breeders could consider using this SNP to select for high or low flavanol content in seedling populations because it is significantly associated with all of the compounds in the 3.0- to 4.0-Mb megahotspot on Chr. 16 (Fig. 3; other compounds not shown). Combining this marker into a haplotype with the other two peak SNPs on Chr. 16 could be a way to further improve selection (Supplementary Fig. S6), however specific test populations would be needed to validate all allele combinations. The Chr. 15 marker *15_3,168,243* could alternately be applied to directly target epicatechin and procyanidin C_1_ without impacting other flavanols. For dihydrochalcones, the marker *5_29,703,521* could be used to select for phloretin glycosides. Importantly, increasing concentrations of dihydrochalcones through selective breeding could help to improve nutritional value in apple fruits without increasing bitterness [56]. All of these candidate markers require validation within segregating populations to assess their true breeding value.

### Metabolite identification

Due to the limited availability of commercial reference standards, confirming the annotation and identification of most unknown features remains a persistent challenge for metabolomics in fruit crops. In the present study, our identifications were supported by both reference standards and previous publications identifying phenolic compounds across several apple varieties using HPLC-NMR-MS [57–59]. In order to improve the identification and characterization of unknown features, we also proposed to utilize genetic information. In cases where a genomic hotspot included reference-standard confirmed compounds, we postulated that other compounds mapped to the same locus could have similar structures and be derived from similar metabolic pathways as the reference standard, based on their similar retention times, masses, and mGWAS results. This consideration was applied in one instance to identify a Chr. 5 hotspot compound: phloretin-2'-O-(2"-O-xylosylglucoside) (Fig. 3). Here, the putative compound identification was enabled by comparing the mGWAS results and mass spectral data for the previously unknown compound (7.43_568.1800n) with those for phloridzin (7.43_436.1370n). Although we were cautious to not overextend this approach, it may become more widely useful for compound identification as phenomic and genomic datasets increase in size and are integrated into larger multi-omics modeling studies or meta-analyses.

Another approach was to collect ion mobility data on all features. For example, an ion of m/z 287.0556 was detected at retention times of 4.53, 5.19, 7.13, and 8.04 min, and with ^TW^CCS_N2_ ion mobilities of 161.27, unknown, 159.18, and 159.18, respectively. The mass with a retention time of 4.53 min mapped to the same genomic position on Chr. 9 as cyanidin 3-O-beta-D-galactoside (4.55_449.1086 m/z), whereas the others had no strong mGWAS peaks (Supplementary Table S4). Ion mobility data allowed this metabolite (4.53_287.0556 m/z) to be differentiated from others of the same mass, and it was annotated as the cyanidin-like compound 5,7-dihydroxy-3-(2,4,5-trihydroxyphenyl)-2,3-dihydro-4H-chromen-4-one (Supplementary Table S3). Although ion mobility data collection and measurement depend on the instrumental setup and resolution [60], as well as on the experimental data available for comparison [61], our proposed identifications were mostly in agreement with previous reports of phenolic compounds [61–63]. These examples demonstrate that considering mGWAS mapping results and ion mobility alongside retention time and mass appears to be a reasonable approach to aid in the annotation of unknown metabolites, especially in the absence of commercial reference standards. To this end, we will continue to analyze the unknown and putatively identified compounds discussed in this study and provide updates as new reference standards become available.

## Conclusion and future directions

Large-scale untargeted metabolomics combined with GWAS has revealed novel genomic hotspots and candidate genes associated with hundreds of health-active phytochemicals in diverse apples. Our results suggest that 3.0–4.0 Mb on Chr. 16 is the most important genomic region controlling phenolic compound abundance in diverse apple germplasm. Peak SNPs in this region could be applied to enable marker-assisted breeding for >200 phenolic compounds. Polymorphisms detected within the bHLH and C2H2 transcription factors in the same region point to these specific genes as playing a central role in the apple phenolic regulatory network. For example, they may control expression of previously reported biosynthesis genes such as *LAR1*. The cluster of oxidoreductase genes detected at another major locus on Chr. 5 may also be participants in the same network.

The apple phenolic compounds controlled by major loci on Chr. 5 and 16 serve important functions in plant growth and development, fruit ripening, and response to the environment. In addition, their antioxidant capacities make them highly nutritious for human consumers. As such, the candidate genes and loci detected in these regions should be further explored to not only better understand the biosynthesis of phenolic compounds in apple, but also to evaluate potential targets of genetic improvement. We therefore aim to continue the work of characterizing and validating the candidate genes and loci that were most strongly associated with flavonoid and dihydrochalcone phenolic compounds. Whether through marker-assisted selection or gene editing, the results of this research will provide new opportunities to breed highly nutritious apples with improved plant health attributes, better eating quality, and potential pharmaceutical applications.

## Materials and methods

### Fruit materials

Apple fruits were harvested from the apple biodiversity collection (ABC), which contains a total of 1119 apple genotypes including *Malus* × *domestica* and *Malus sieversii* accessions and is located at the Kentville Research and Development Centre, Agriculture and Agri-Food Canada (AAFC) in Nova Scotia, Canada (45.071767, −64.480466). Detailed information about the ABC was reported previously [24]. Briefly, this collection includes modern cultivars, heritage varieties, numbered selections from breeding programs, and wild genebank accessions. Germplasm within the collection originated primarily in the USA and Canada, followed by Asia and Europe, with years of origin ranging from the 1700s to present day. The orchard is grown in a temperate climate with daily average temperatures ranging from −5.6°C in January to 19.5°C in July and 1181 mm of average annual precipitation (https://climate.weather.gc.ca/climate_normals/). The orchard is planted in an experimental design with two replicates per genotype; the replicates were separated into North and South blocks each with 1119 genotypes [24].

For the purposes of this study, 439 accessions were harvested and evaluated in the fall of 2016. This represented the subset of the ABC for which ripe fruits could be harvested and evaluated in 2016; the 349 apples capture the full range of genetic variation present in the larger collection (Supplementary Fig. S2). Fruit maturity was determined by ground color, firmness assessed by touch, sweetness assessed by taste, browning of seeds, and starch-iodine content [64]. Fifteen fruits were harvested from each sampled accession and stored at 3°C for 30 days. Basic fruit quality traits including size, firmness, soluble solids content, juice content, and titratable acidity were previously evaluated and reported by Watts *et al*. (2021) [14]. Peel and flesh tissues from 10 to 15 apples per sample were frozen in liquid nitrogen, then ground into powder and stored at −80°C for analysis in the present study.

### Metabolomics analysis

#### Chemicals

All chemical solvents as HPLC grade were purchased from Fisher Scientific (Georgetown, ON) and Waters (Waters Corporation, Milford, MA, USA). Reference standard compounds including caffeic acid, chlorogenic acid, catechin, epicatechin, cyanidin 3-galactoside, quercitrin, avicularin, phloretin, phloridzin, and rutin were purchased from Sigma-Aldrich Chemical Co. (St. Louis, MO). Procyanidins A_2_, B_1_, B_2_, and C_1_ were purchased from Indofine Chemical Co. (Hillsborough, NJ). Isoquercitrin was purchased from Fluka Chemie GmbH (Buchs, Switzerland).

#### Sample extraction and separation

The sample extraction protocol was performed as previously reported [65]. Briefly, 0.5 g tissue was extracted twice with 0.7 ml of extraction solvent (80:20 methanol: water, V/V, with 0.1% formic acid) followed by mixing and sonication. The extract was centrifuged, then the supernatants from the two extractions were combined and dried in a vacuum centrifuge (Thermo Fisher). The extracts were then redissolved in 1 ml solution (10% methanol with 0.1% formic acid) and sonicated, vortexed, and centrifuged. The supernatants were diluted and transferred to HPLC vials for injection. A reference compound, Telmisartan [66] [M + H]^+^ m/z 515.2444 at concentration of 250 ng ml^−1^ was added to each sample as an internal standard. A NanoAcquity UPLC system (Waters Corporation, Milford, MA, USA) equipped with a BEH C_18_ 1.7 μm 1.0 × 100 mm column (Waters Corporation, Milford, MA, USA) was used to conduct LC separation. Two extractions were conducted on each accession to serve as technical replicates. LC analysis performed as previously reported [65].

#### Mass spectrometry

Untargeted metabolomics analysis was conducted as previously reported with slight modifications [65]. The MS analysis was conducted on a Q-TOF mass spectrometer (Synapt XS, Waters Corporation, Milford, MA, USA) equipped with an electrospray ionization source (ESI). Data acquisition was performed using data-independent acquisition (MS^e^) mode in the continuum mode using MassLynx (version 4.2, Waters Corporation, Milford, MA, USA). The MS^e^ acquisition parameters were set as previous [65]. The mass detector was operated in both positive and negative modes at high resolution of 40 000 (FWHW at m/z 556.2771). Leucine enkephalin ([M + H]^+^ m/z: 556.2771) was used as a lock mass solution. A standard mixture of 15 reference compounds (listed under Chemicals) was also prepared and run as quality control to assist with identification based on retention time, mass accuracy, and mass fragmentation. A matrix sample containing 100 random selected samples of equal amount was prepared and injected five times at the beginning of each run of 90 samples, plus once between every 10 samples to minimize variation of the analysis [67].

We also conducted ion mobility analysis with the Traveling Wave ion mobility setting to determining the collisional cross section (^TW^CCS_N2_) values of reference standard and feature QC-matrices in positive mode to facilitate feature classification and identification (Supplementary Method SM1).

#### Data processing and analysis

Progenesis QI (Version 3.0, Waters Corporation, Milford, MA) was employed to process and analyze the raw MS data by detecting masses of peak groups to align the low-energy and high-energy data as well as retention alignment, adducts, and deconvolution. Results were generated with all detected masses (m/z), retentions time, and integrated intensities normalized against the internal standard using Progenesis QI. The detected mass and RT values are referred to as features, and feature abundances are reported in detector response units (DRU). For each feature, a single-factor linear variance analysis was performed on DRU values considering the effects of apple genotype (*n* = 2) to estimate the mean abundance for each genotype. The parameters for Progenesis QI were as following: sensitivity level for peak picking as default, minimum chromatographic peak with 0.20 min, RT range, 2.0–13.0 min.

#### Metabolite identification

Identification of metabolites was conducted using Progenesis QI (Version 3.0) as in [65]. Online databases such as Chemspider (chemspider.com), FoodDB (foodb.ca), and KEGG (genome.jp/kegg/compound) were used to provide annotations of the features. Mass tolerances for MS and MS/MS were ±5.0 ppm and the threshold for isotope similarity was 90%. Manual inspection of MS/MS fragmentation spectra, mass accuracy verification, isotope analysis, and searching of publicly available databases were applied. The elemental composition calculator of MassLynx 4.2 was also used to determine the elemental composition of compounds. After manual inspection, only peaks with a Progenesis QI score >39 and fragment score and variation of mass error <±5.0 ppm were considered as tentative identifications and confirmed with authentication standards for retention times and m/z when possible. The metabolites were annotated according to the metabolomics standard initiative guidelines and recommendations [68]. A checklist for the experiment setup and LC–MS metabolomic study is summarized in Supplementary Table S1**.**

### Metabolomic GWAS

#### Genotypic data

The genotypic data used in this study were retrieved from [69]. The preliminary dataset consisted of 278 231 SNPs derived from genotyping by sequencing and imputation for 1175 diverse apple accessions in the ABC. The SNPs were evenly distributed across the apple genome with no missing data and provided saturating coverage required to detect all major QTL [69]. The same SNP panel was used in previously published GWAS for fruit quality traits [24] and aroma volatiles [70]. For the present study, the SNP panel was filtered using TASSEL [71] firstly to include only the apples for which both metabolomic and genotypic data were available, and secondly to a minimum 1% minor allele frequency. The resulting dataset consisted of 270 440 genome-wide SNPs covering 439 apple accessions.

#### Genome-wide association study

The mGWAS was performed using a mixed linear model (MLM) analysis controlling for variance due to family-based kinship (K) and cryptic (Q) relationships among apple genotypes [71]. The K- and Q-matrices were estimated using the default parameters in TASSEL, respectively centered-IBS with maximum six alleles and covariance with five components. Separate MLM analyses were run for each of the 2066 metabolites identified in the positive ion mode UPLC–MS. Significant associations were identified based on a Bonferroni-corrected *P*-value of 1.85 × 10^−7^. Visual inspection of Manhattan plots qq plots was used to identify strong peaks based on the MLM and PCA kinship, which were characterized as a columnar to narrow triangular stack of markers with the peak marker surpassing the significance threshold. Manhattan plots with scattered markers and/or qq plots suggesting weak model fit were removed from consideration. These conservative thresholds (Bonferroni correction plus visual triage) were applied in order to focus the analysis on the strongest associations with the most likely biological relevance to phenolic content in apple. Manhattan plots were visualized using packages tidyverse [72] and qqman [73] in R 4.2.

#### Marker–trait associations

The most significant SNPs from each strong peak were aggregated for all metabolites (Supplementary Table S4). Markers that were the most significant SNP for three or more metabolites were identified as hotspots. The genomic positions of significant SNPs relative to gene features were classified with a custom R script (Supplementary Method SM3). For hotspot SNPs within coding regions, the precise location and codon sequence were examined manually in the GDDH13 v1.1 reference genome [27] using J-Browse hosted by the Genome Database for Rosaceae (GDR) [25] and the allele differences and corresponding differences in nucleotide codons and amino acids were obtained. Boxplots of compound abundances versus SNP alleles were visualized using tidyverse [72] in R 4.2.

#### Phenotypic analyses

Phenotypic distributions for the mass features detected in positive mode were assessed using density plots drawn with the R packages tidyverse [72] and ggridges [74]. A PCA was performed to identify multivariate relationships among apples based on the positive mode metabolite abundances using the PCA function of R package FactoMineR with scaled variables. A phenotypic correlation analysis was performed between all reference standards and putatively identified metabolites for which strong peaks were identified in the mGWAS analysis. Pairwise Pearson correlations were estimated using the *rcorr* procedure of the R package Hmisc [75] with a Bonferroni correction applied at α = 0.05. The PCA biplot and heatmap of correlation coefficients were visualized using tidyverse [72].

## Supplementary Material

Web_Material_uhaf159

## Data Availability

Supplementary information accompanies the manuscript in the supplemental tables and files provided. This paper makes use of genotypic data that were previously published and are available online [69].
