# Supplementary material for: Untargeted metabolomic genome-wide association study reveals genetic and biochemical insights into polyphenols of apple fruit
Source: Hortic Res. 2025 Aug 12;12(9):uhaf159. doi: 10.1093/hr/uhaf159 (PMC12377893; doi:10.1093/hr/uhaf159)

Supplementary Figures

Untargeted metabolomic genome-wide association study reveals genetic and biochemical insights into polyphenols of apple fruit

Jun Song, Beatrice Amyotte Leslie Campbell Palmer, Melinda Vinqvist-Tymchuk, Kyra Dougherty, and Letitia Da Ros[2]

Supplementary Figure SF1. (a) PCA biplot of apple varieties based on metabolite abundance with dots coloured according to cumulative metabolite abundance and varieties with highest and lowest values circled. (b) Density plots showing phenotypic distributions of cumulative metabolite abundance and reference standard metabolite abundance across apple population.

Supplementary Figure SF2. Genomic PCA for Apple Biodiversity Collection of 1119 accessions, indicating (a) the subset represented in this mGWAS and (b) the Malus species represented. Principle component data for 278,000 genome-wide SNPs obtained from Migicovsky et al. 2022 (https://doi.org/10.3389/fgene.2022.934712)

Supplementary Figure SF3. Clustering of chemical compounds. (a) Dendrogram for all metabolomic features detected in the population with putatively identified compounds marked in red. (b) Dendrogram for selected features detected as group of flavanols (epicatechin and procyanidin-related compounds). These features share a significant GWAS hit on Chromosome 16 at position 3,421,803, indicating a close chemical and genetic relationship. There was no significant GWAS hit for catechin. (c) Dendrogram for selected features detected as group of dihydrochalcones. These features share significant GWAS hits on Chromosome 5 at position 29,703,521 and 29,721,457. Clustering performed using default parameter in Progenesis QI (Waters, https://www.nonlinear.com/progenesis/qi/).

Supplementary Figure SF4. Reproducibility of LC-MS analysis of selected reference standard compounds (catechin and epicatechin) at concentration of 0.13 µmole mL^{-1} and 0.12 µmole mL-1 36 for 60 injections from all batches (6 plates) through this study. The average of RSD for catechin is 4.78 % and epi-catechin is 2.3%. The detailed experiment design and LC-MS setting can be found in M & M section and supplementary Table S1.

Supplementary Figure SF5. Linkage disequilibrium on Chr 16 (a) overall and (b) within mGWAS hotspot at 3-4 Mb. LD estimated in TASSEL using default parameters (heterozygous markers ignored).

Supplementary Figure SF6. Phenotypic means for Procyanidin B1 (F0373) and ranges by three-marker haplotype from Chr 16. Different letters indicate significant differences between means according to a Tukey's HSD Test ($p<0.05$). Haplotypes not listed have n = 0.

SF1

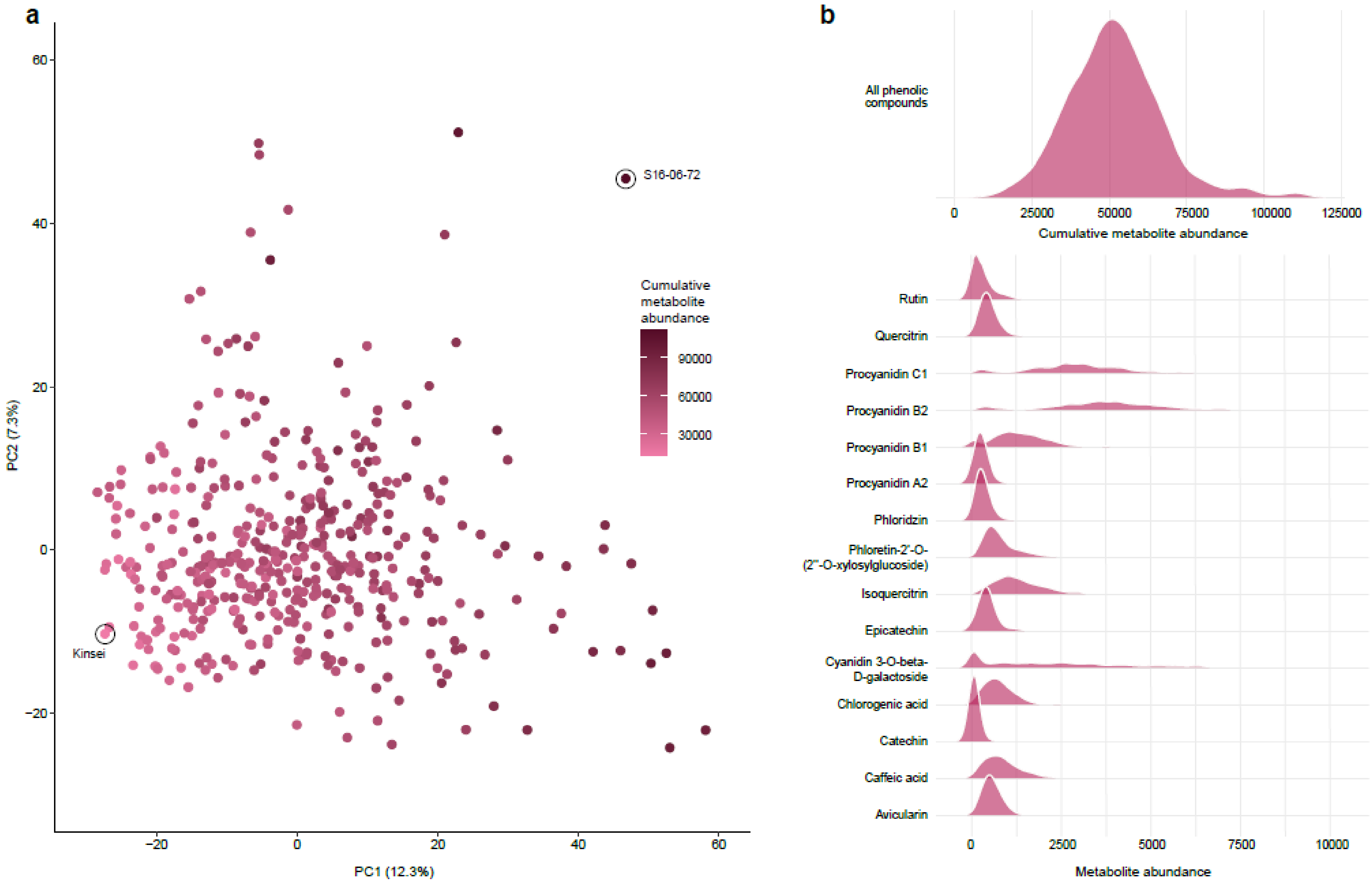
a
S16-06-72
Kinsei
Cumulative metabolite abundance
90000
60000
30000
60
40
20
0
−20
PC2 (7.3%)
−20
0
20
40
60
PC1 (12.3%)
b
All phenolic compounds
0
25000
50000
75000
100000
125000
Cumulative metabolite abundance
Rutin
Quercitrin
Procyanidin C1
Procyanidin B2
Procyanidin B1
Procyanidin A2
Phloridzin
Phloretin-2'-O-(2'''-O-xylosylglucoside)
Isoquercitrin
Epicatechin
Cyanidin 3-O-beta-D-galactoside
Chlorogenic acid
Catechin
Caffeic acid
Avicularin
0
2500
5000
7500
10000
Metabolite abundance

SF2

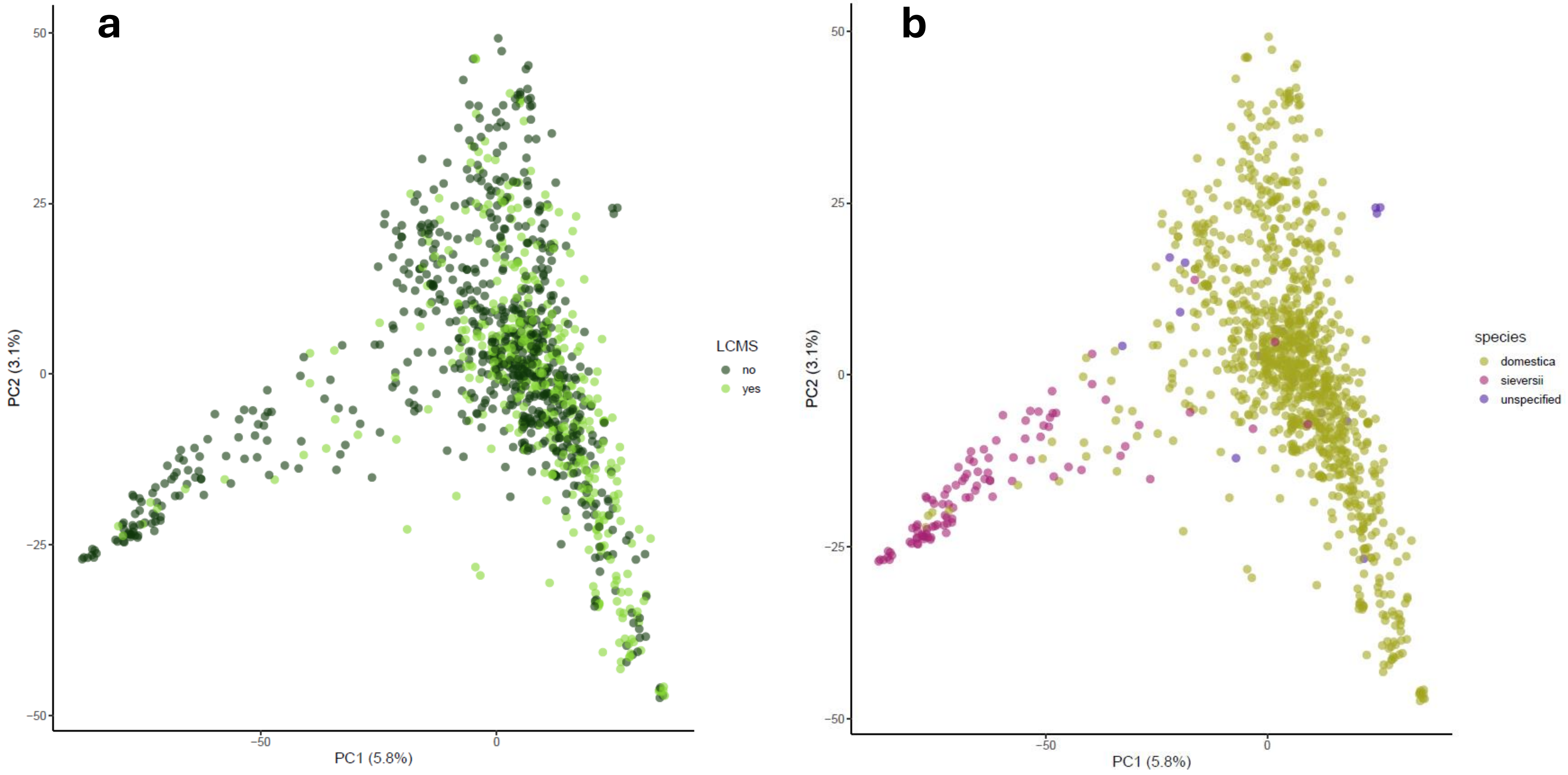
a
50
25
0
-25
-50
-50
0
PC1 (5.8%)
PC2 (3.1%)
LCMS
no
yes
b
50
25
0
-25
-50
-50
0
PC1 (5.8%)
PC2 (3.1%)
species
domestica
sieversii
unspecified

SF3

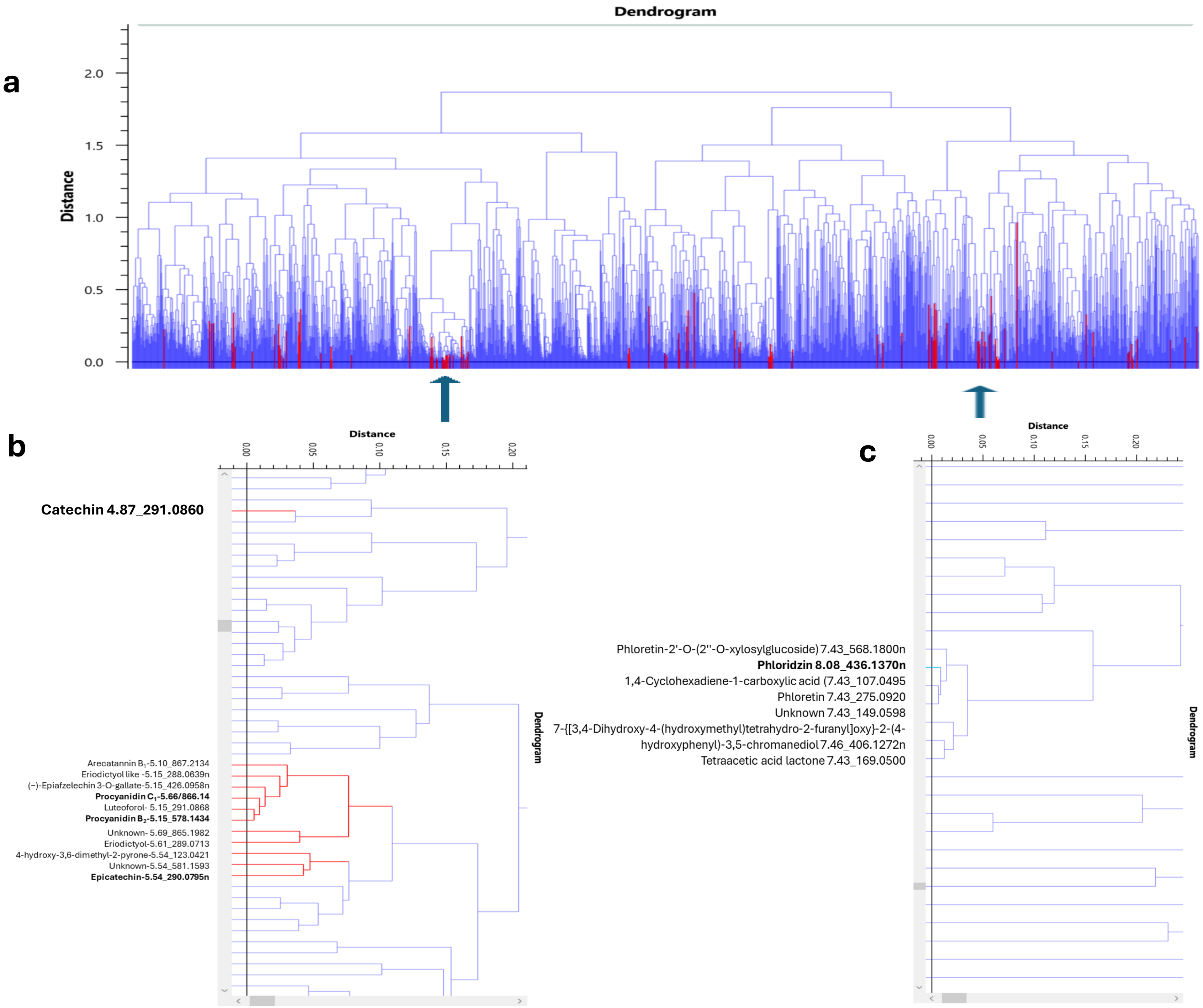
a
Dendrogram
Distance
0.0
0.5
1.0
1.5
2.0
b
Distance
0.00
0.05
0.10
0.15
0.20
Catechin 4.87_291.0860
Arecatannin B1-5.10_867.2134
Eriodictyol like -5.15_288.0639n
(−)-Epiafzelechin 3-O-gallate-5.15_426.0958n
Procyanidin C1-5.66/866.14
Luteoforol- 5.15_291.0868
Procyanidin B2-5.15_578.1434
Unknown- 5.69_865.1982
Eriodictyol-5.61_289.0713
4-hydroxy-3,6-dimethyl-2-pyrone-5.54_123.0421
Unknown-5.54_581.1593
Epicatechin-5.54_290.0795n
Dendrogram
c
Distance
0.00
0.05
0.10
0.15
0.20
Phloretin-2'-O-(2''-O-xylosylglucoside) 7.43_568.1800n
Phloridzin 8.08_436.1370n
1,4-Cyclohexadiene-1-carboxylic acid (7.43_107.0495
Phloretin 7.43_275.0920
Unknown 7.43_149.0598
7-{[3,4-Dihydroxy-4-(hydroxymethyl)tetrahydro-2-furanyl]oxy}-2-(4-hydroxyphenyl)-3,5-chromanediol 7.46_406.1272n
Tetraacetic acid lactone 7.43_169.0500
Dendrogram

SF4

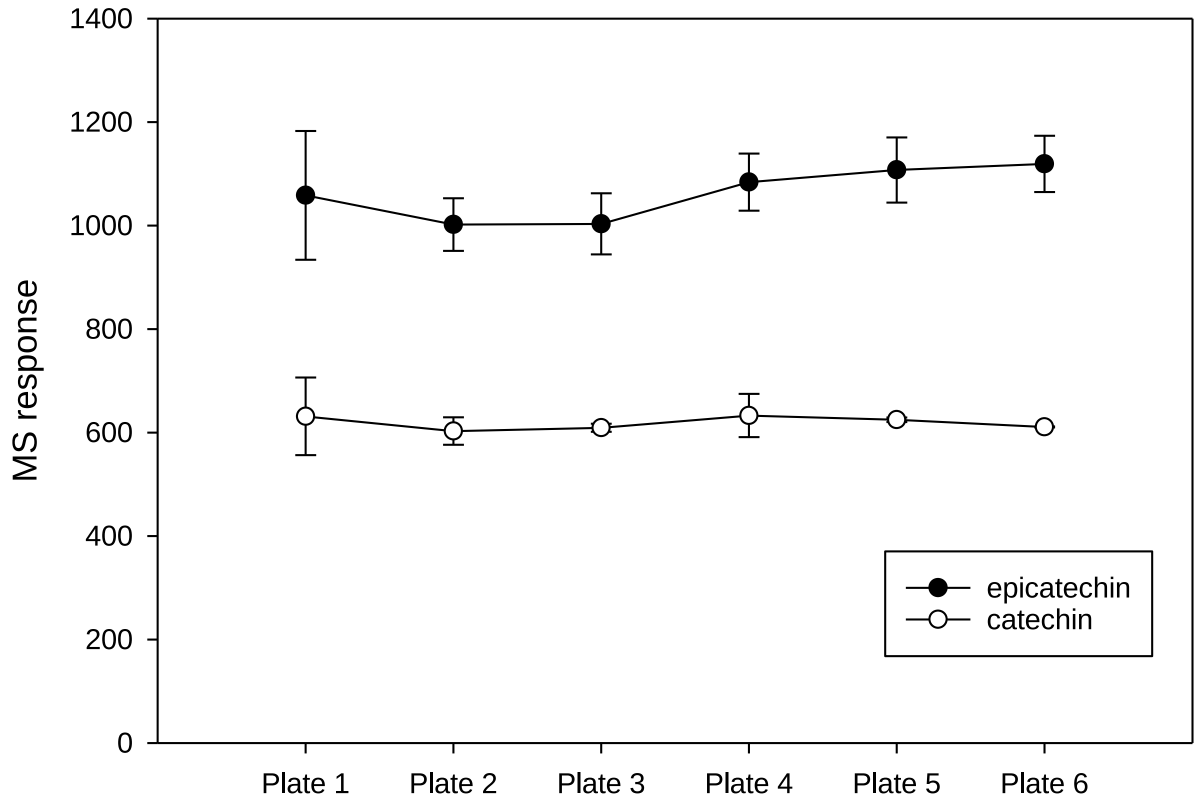

MS response
1400
1200
1000
800
600
400
200
0
Plate 1
Plate 2
Plate 3
Plate 4
Plate 5
Plate 6
epicatechin
catechin

SF5

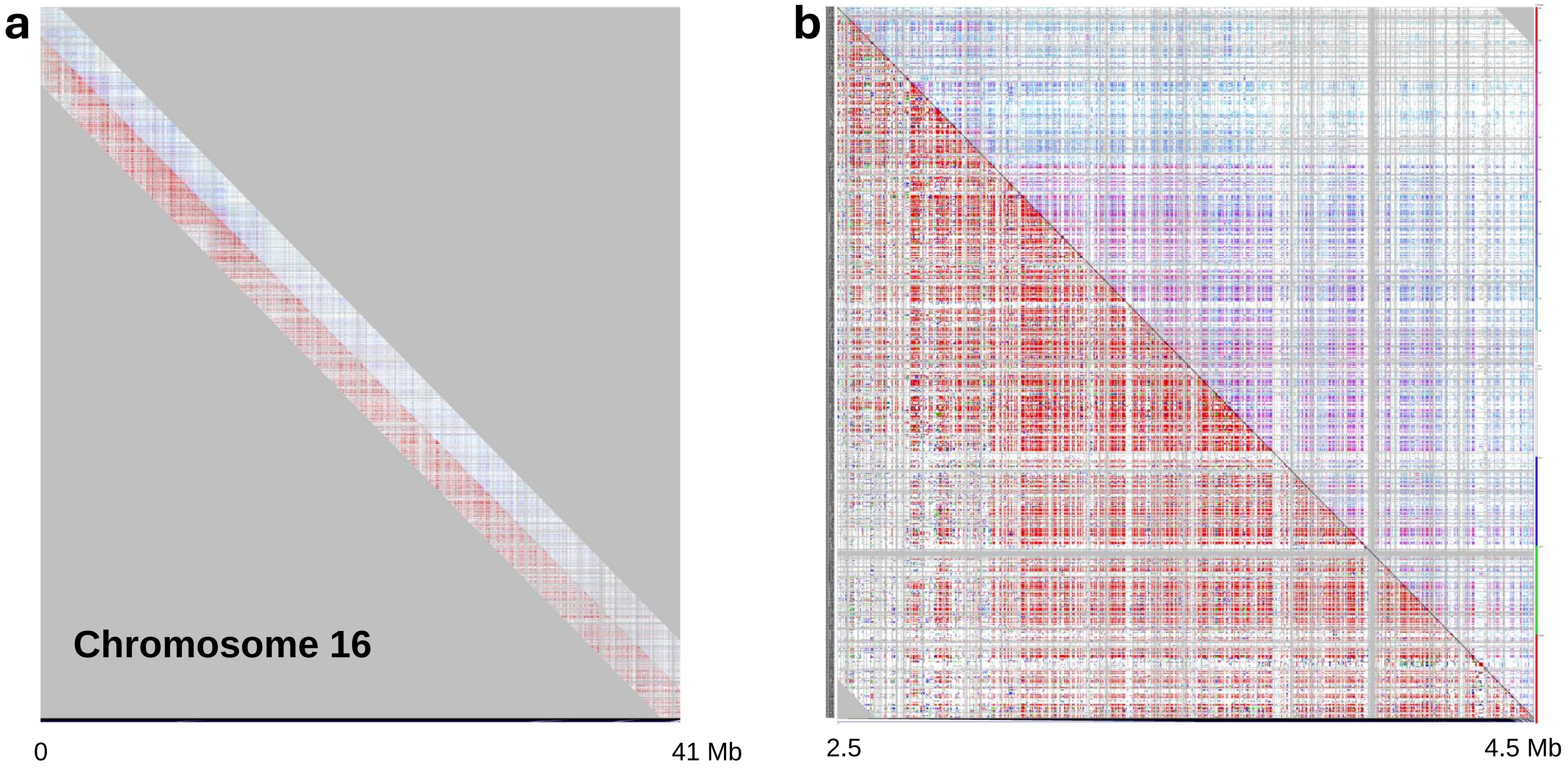

SF6

Haplotype Ch16 3,178,627; 3,304,530; 3,421,803;	n	Procyanidin B1 (F0373) Mean abundance	Example Accessions	Tukey Group
A;C;T	45	2027	Grise Dieppoise (890) American Forestier (436)	a
A;C;Y	2	-		-
A;M;Y	2	-		-
G;A;T	1	-		-
G;A;Y	2	-		-
G;A;C	36	192	Zestar (56) Kinsei (841)	c
G;M;T	1	-		-
G;M;Y	20	1371	Karastojanka (775) Milwaukee (1230)	b
G;M;C	3	-		-
G;C;T	3	-		-
G;C;Y	4	-		-
R;A;C	2	-		-
R;M;T	6	-		-
R;M;Y	258	1327	Beauty of Bath (461) Gladstone (746)	b
R;M;C	6	-		-
R;C;T	24	1765	Erickson (756) S47-21-84 (628)	ab
R;C;Y	24	1590	Kaz 95 18-02P-20 (108) Rose de Benauge (1104)	ab

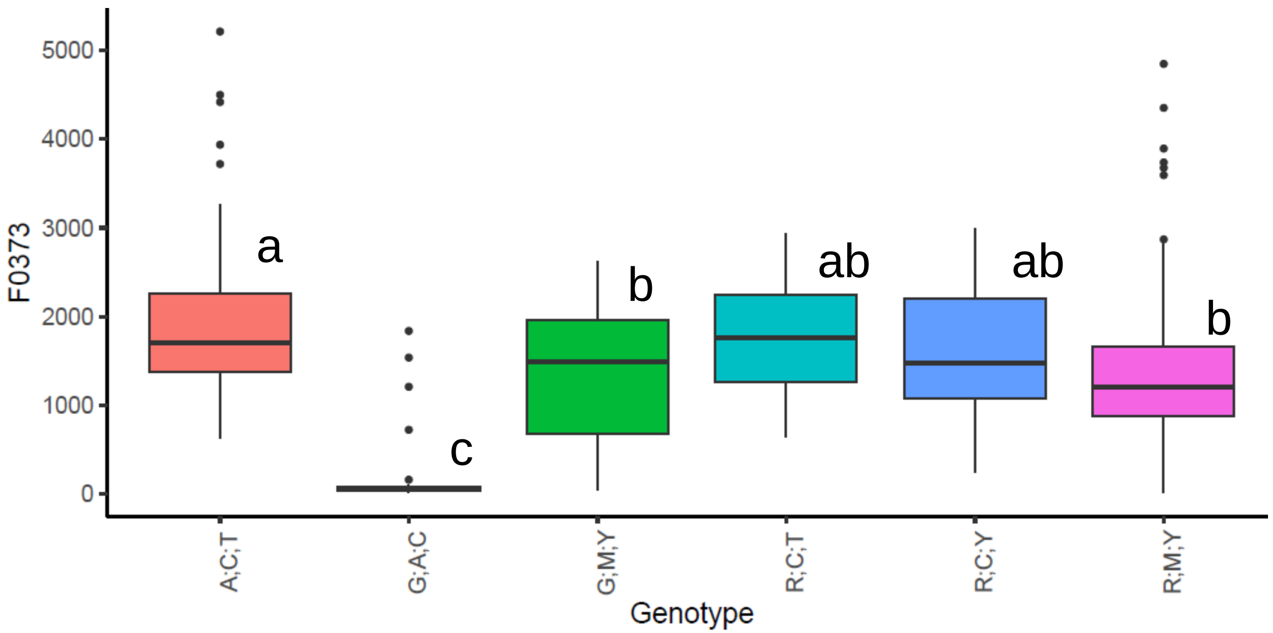

Supplement: Web_Material_uhaf159 [file web_material_uhaf159.zip › Supplementary Figures [2025-04-28].pdf]
