# Supplementary material for: Untargeted metabolomic genome-wide association study reveals genetic and biochemical insights into polyphenols of apple fruit
Source: Hortic Res. 2025 Aug 12;12(9):uhaf159. doi: 10.1093/hr/uhaf159 (PMC12377893; doi:10.1093/hr/uhaf159)

**Supplementary Method Figure SM2**. Overall relationship between detected ions (RT and mass) of apple matrix sample with drift time detected using Synapt HDMS IM-QTOF mass spectrometry.


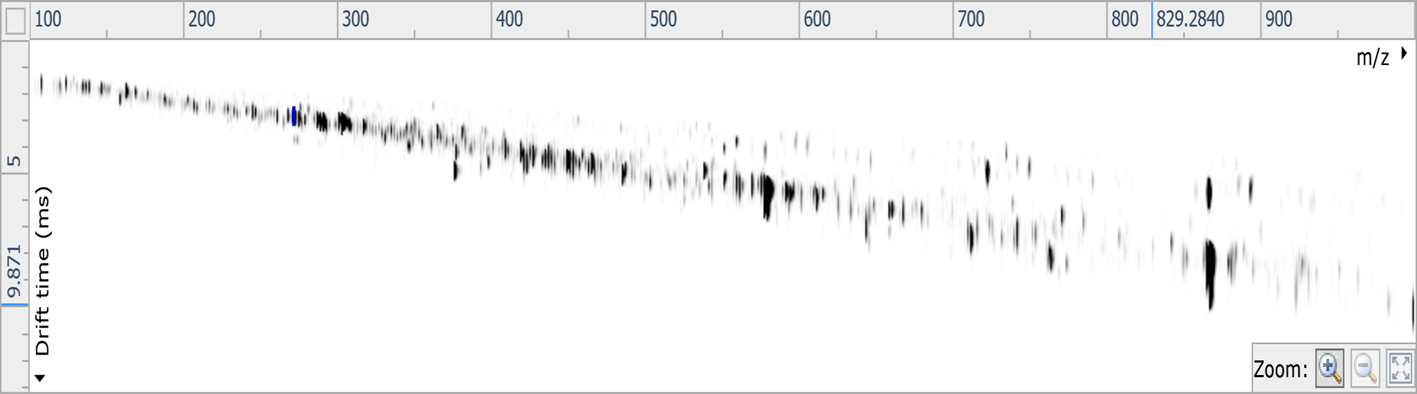

Supplement: Web_Material_uhaf159 [file web_material_uhaf159.zip › Supplementary Method Figure SM2.docx]
