# Supplementary material for: Untargeted metabolomic genome-wide association study reveals genetic and biochemical insights into polyphenols of apple fruit
Source: Hortic Res. 2025 Aug 12;12(9):uhaf159. doi: 10.1093/hr/uhaf159 (PMC12377893; doi:10.1093/hr/uhaf159)
Supplement: Web_Material_uhaf159 [file web_material_uhaf159.zip › Supplementary Method SM1-Traveling wave ion mobility.docx]

**Supplementary Method SM1**

Calibration and measurement of ^TW^CCS_N2_ value using Synapt HDMS IM-QTof mass spectrometry.

Traveling wave ion mobility (TWIMS) was conducted on the same MS as described in Materials and Methods in positive mode except for the ion mobility (IM) setting. The procedure for ion mobility settings is as follows: sampling con 35 V, helium cell gas 180 ml min^-1^, IM buffer gas (N_2_) with flow rate 90 ml min^-1^, IM velocity 700 m s^-1^ and height 40 V, trap wave velocity 311 m s^-1^ and height 6 V. IM standards mixture (ref. 186008113, Waters, Millford, US ) was used as calibration standard to determine the ^TW^CCS_N2_ value in positive mode. It covers the mass range of 50 to 1,200 Da and CCS value range from 152- to 309 A^2^. The calibration curve was established at R^2^ =0.9997 (**Supp. Table S5, Fig. SM2**). The matrix and reference standard samples were also analyzed under the IM-MS condition and repeated three times. The average of the measurements was presented to assist the characterization and identification of the samples. The data analysis was conducted using Progenesis QI.
